# Fast and Accurate Discovery of Degenerate Linear Motifs in Protein Sequences

**DOI:** 10.1371/journal.pone.0106081

**Published:** 2014-09-10

**Authors:** Abdellali Kelil, Benjamin Dubreuil, Emmanuel D. Levy, Stephen W. Michnick

**Affiliations:** Département de Biochimie and Centre Robert-Cedergren, Bio-Informatique et Génomique, Université de Montréal, Succursale Centre-Ville, Montreal, Quebec, Canada; University of Toronto, Canada

## Abstract

Linear motifs mediate a wide variety of cellular functions, which makes their characterization in protein sequences crucial to understanding cellular systems. However, the short length and degenerate nature of linear motifs make their discovery a difficult problem. Here, we introduce MotifHound, an algorithm particularly suited for the discovery of small and degenerate linear motifs. MotifHound performs an exact and exhaustive enumeration of all motifs present in proteins of interest, including all of their degenerate forms, and scores the overrepresentation of each motif based on its occurrence in proteins of interest relative to a background (e.g., proteome) using the hypergeometric distribution. To assess MotifHound, we benchmarked it together with state-of-the-art algorithms. The benchmark consists of 11,880 sets of proteins from *S. cerevisiae*; in each set, we artificially spiked-in one motif varying in terms of three key parameters, (i) number of occurrences, (ii) length and (iii) the number of degenerate or “wildcard” positions. The benchmark enabled the evaluation of the impact of these three properties on the performance of the different algorithms. The results showed that MotifHound and SLiMFinder were the most accurate in detecting degenerate linear motifs. Interestingly, MotifHound was 15 to 20 times faster at comparable accuracy and performed best in the discovery of highly degenerate motifs. We complemented the benchmark by an analysis of proteins experimentally shown to bind the FUS1 SH3 domain from *S. cerevisiae*. Using the full-length protein partners as sole information, MotifHound recapitulated most experimentally determined motifs binding to the FUS1 SH3 domain. Moreover, these motifs exhibited properties typical of SH3 binding peptides, e.g., high intrinsic disorder and evolutionary conservation, despite the fact that none of these properties were used as prior information. MotifHound is available (http://michnick.bcm.umontreal.ca or http://tinyurl.com/motifhound) together with the benchmark that can be used as a reference to assess future developments in motif discovery.

## Introduction

Linear motifs in proteins play key roles in molecular recognition [1]–[3]. They mediate diverse functions including ion-coordination [4], protein localization [2], [5], protein cleavage [2], protein assembly through scaffolding [1], [2], [6], [7], protein post-translational modifications [2], [5], or more generally signal transduction [1]. The rich functional repertoire of linear motifs is also well illustrated in their extensive use by viruses to hijack the machinery of host cells [8], [9]. Typically, linear motifs (LMs) conform to a particular sequence pattern (i.e. a consensus sequence), where certain residues are constrained in their amino acid identity (e.g., “P” in PxxP), whereas others are not (e.g. “x” in PxxP), and are also called wildcards. In this work, we use the term “degenerate” to refer to motifs containing wildcard positions. Linear motifs are typically 3 to 10 amino acids long, though only few residues (∼1/3) are generally conserved due to their importance in motif recognition [10], [11]. Such short length and degenerate nature make their discovery a difficult problem, yet, their functional importance and widespread nature stresses the need for methods to help in their ab-initio discovery.

Over the past three decades, many computational approaches have been developed to predict functional LMs. In Tables **Table 1**, we summarize these methods and some of their key features. Schematically, *ab-initio* motif search can be decomposed into two steps; the first step consists in searching (*e.g.* enumerating or sampling) the candidate motifs present in protein sequences of interest, while the second step consists in scoring the candidate motifs in order to assess their biological significance.

**Table 1:** Algorithms used in this study to predict linear motifs and motif-rich regions.

Algorithm	Description	Advantages	Limits	code/webserver	used in the benchmark
**MotifHound**	Exhaustively finds motifs overrepresented in a given set of sequences relative to background sequences (e.g., an entire proteome)	Fast; Multi-thread version available; Exhaustive exploration of motifs; Can work with filters (disorder, conservation)	Requires a minimum of three input sequences and a background; RAM-expensive	Yes/No	Yes
**DILIMOT [12]**	Finds overrepresented motifs relative to random sequences taken from SWISSPROT	Integrates several types of sequence information on motifs (e.g., disorder, conservation)	Source code not available	No/Yes	No (code NA)
**SLiMFinder [14]**	Finds overrepresented motifs relative to a background model of sequences	Well documented; Can work with filters (disorder, conservation)	Fixed motif length cannot be set; CPU-expensive	Yes/Yes	Yes
**MEME [28]**	Explores the motif space using Gibbs sampling and expectation maximization	Fast, Multi-thread; Eye friendly output	Output difficult to parse; Heuristic solution not guaranteed to be optimal	Yes/Yes	Yes
**TEIRESIAS [13]**	Finds motifs that are frequent in a dataset of interest relative to a background model of sequences	Very fast	Motif length cannot be set; Can miss wildcard rich motifs	Yes/Yes	Yes
**FIRE-pro [60]**	Uses k-mers exploration to find motifs overrepresented in sequences of interest relative to background sequences.	User friendly output	CPU-expensive; Can miss wildcard rich motifs	Yes/Yes	No (running time)
**NESTEDMICA [16]**	Uses nested sampling to find motifs overrepresented in sequences of interest relative to a background model.	Models motifs as position weighted matrices	CPU-expensive; Heuristic solution not guaranteed to be optimal	Yes/Yes	No (running time)
**qPMS7 [62]**	Find motifs overrepresented in sequences of interest based on Quorum Planted Motif Search	Fast; Low memory consumption	Source code limited to 20 protein sequences; Output difficult to parse	Yes/Yes	No (limited to 20 protein sequences)
**D-STAR [63]**	Finds motifs enriched in specific protein interaction partners	Well-suited to find motifs shared among interaction partners	CPU-expensive; Interaction file required to run the program	Yes/No	No (running time)
**phylo-HMM[21]**	Detects evolutionarily conserved regions from aligned protein sequences using a phylogenetic Hidden Markov Model (HMM)	-	-	Yes/No*	No (predicts regions not motifs)
**ANCHOR [20]**	Predicts regions containing linear motifs based on sequence properties.	-	-	Yes/Yes	No (predicts regions not motifs)
**SLIMPRED [61]**	Predicts regions containing linear motifs based on a trained neural network	-	-	No/Yes	No (predicts regions not motifs)

*Provides pre-computed results for *S. cerevisiae and H. sapiens* but does not accept sequences for submission.

The biological significance of linear motifs is typically assessed by their “unexpectedness”, which is influenced by their sequence, by their enrichment in specific proteins, by their complexity (*i.e.* number of different amino acids) and by their length [12]–[16]. In other words, a motif made up of rare amino acids is more significant than a motif made up of frequent amino acids; and a motif enriched in a specific group of proteins is more significant than a motif present in a single protein. Biologically relevant linear motifs often exhibit a statistical significance that lies in or near the twilight zone (*i.e.* where there is a non-negligible probability to observe a random motif) [17]. This difficulty begs for a better understanding and characterization of motif statistical significance [2], [18], and calls for alternative approaches. On top of these statistical considerations, additional filters such as evolution and disorder have been used to pinpoint motifs most likely to be functional [19]–[21]. It was indeed recently estimated that at least 5% of amino acids in disordered regions are important for function [21]. Different strategies have been employed for searching and scoring motifs as briefly described below and summarized in Table 1. DILIMOT [12] is among the first methods designed to tackle the problem of *ab initio* computational discovery of LMs in proteins considering both their overrepresentation and their conservation [21]. Because motifs are enriched in disordered regions [22], DILIMOT removes globular regions and coiled coil regions using information obtained from SMART [23], Pfam [24] and GlobPlot [25]. Regions of strong homology are also filtered, thereby enriching for motifs that have evolved through convergent evolution. Finally DILIMOT uses the pattern search algorithm TEIRESIAS [13] to return raw motifs and ranks them according to conservation and overrepresentation. The latter calculation requires a background probability for finding the motif within randomly selected and similarly filtered sequences from SwissProt [26].

SLiMFinder [14] is a probabilistic LM discovery algorithm that uses a modified version of the TEIRESIAS algorithm called SLiMBuild, allowing to better search for motifs that contain only a small number of defined positions. Proteins can be masked to exclude non-conserved residues, globular regions, low complexity regions, specific amino acids or motifs, and annotated features including domains or user-annotated regions to allow any contextual information to be included in the analyses. Motif significance is calculated using a binomial distribution introduced by ASSET [27] with two major extensions: (1), homologous proteins are weighted to account for the dependencies introduced into the probabilistic framework and (2), significance scores obtained from a binomial distribution are adjusted to take into consideration the size of the theoretical motif space.

Other algorithms, as implemented in MEME [28] or NestedMica [16], are based on probabilistic models that use sampling methods (Gibbs sampling [29] for MEME and Nested sampling [30] for NestedMica) to search statistically overrepresented motifs. Both algorithms use a background model in the form of an *n* ^th^-order Markov chain, and both can be applied to DNA as well as to protein sequences.

Here we introduce a new approach for searching motifs and scoring their biological significance in proteins. In the first step, we exhaustively enumerate all possible motifs present in proteins of interest, including all of their degenerate forms. In the second step, we evaluate the biological significance of the motifs obtained by comparing the number of occurrences of each motif in proteins of interest *versus* in the proteome, which is used as background. Our approach is based on the fundamental premise that, linear motifs mediating a particular function are enriched in proteins exhibiting that function, and are rare or absent in other proteins. In essence, our strategy is comparable to that employed by FIRE-Pro [31] with the significant advantage that MotifHound considers all possible peptide variations as well as all degenerate motifs (*i.e.* it considers wildcards in all numbers and all combinations of positions). In MotifHound, the search is exhaustive (*i.e.* it enumerates all possible motifs present in proteins of interest), and the scoring step is achieved by measuring the enrichment of all enumerated motifs. An advantage of using the proteome as background is that it inherently accounts for any intrinsic structure of the sequence space that would otherwise require complex models such as high-order Markov chains. For example, a Markov model of order three would be needed to model the fact that the motif “SSSS” (present in 601 out of 5761 sequences from *S. cerevisiae*) is seen more frequently than expected by the product of the individual amino acids frequencies (the frequency of S is ∼9%). However, MotifHound would naturally associate a low significance to this motif because it is frequent in the proteome.

Considering that several motif-detection algorithms were previously developed, it may seem surprising that no comprehensive benchmark is available as a point of reference. In order to evaluate the performance of MotifHound and compare it with existing algorithms for motifs discovery, we designed a benchmark as follow. We randomly selected sequences from the proteome of *S. cerevisiae* to create groups of 100 proteins. Within each group, we subsequently spiked-in an arbitrary motif that could vary according to the three following parameters: the number of occurrences spiked-in (*N*), the number of non-wildcards positions (*i.e.* also called number of defined positions *d* that is complementary to the number of wildcard positions *w*) and the motif length *n*. We then tested the performance of each algorithm in the blind identification of the motifs spiked-in. Below we present the results obtained by the different algorithms in terms of both accuracy (*i.e.* capacity to correctly identify the arbitrary motifs randomly inserted into protein sequences), and the run times. We show that our strategy, implemented in MotifHound, yields both accurate results and fast execution times, especially for highly degenerate motifs. In order to provide a biological context for the results of the benchmark, we also analyzed how the three parameters (length *n*, number of occurrences *N* and the number of non-wildcards positions *d*) relate to known motifs extracted from three standard sources (Eukaryotic Linear Motif Resource (ELM) [32], Human Protein Resource Database (HPRD) [33] and MiniMotif [34]). Finally, we present a case study of proteins known to bind to the same SH3 domain, and show that MotifHound can detect most of the known binding motifs using only the full-length sequences as input.

## Materials and Methods

**Figure 1:**
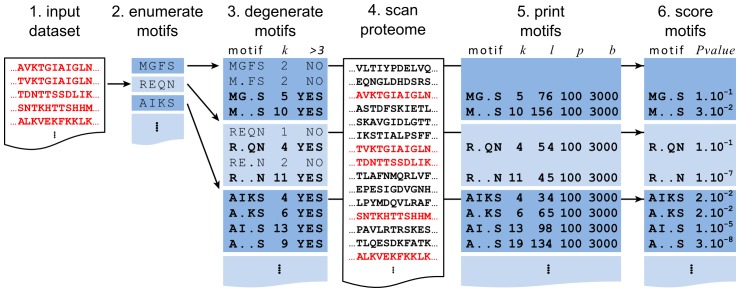
Schematic description of the processing steps involved in motif discovery with MotifHound. (1) Given a set of protein sequences, (2) we enumerate all possible n-mers or motifs (3≤n≤12) present in this query dataset, (3) we then enumerate all degenerate forms of each motif, and we discard those present in less than 3 sequences of the query set. (4) All the motifs retained are counted in the proteome used as background. Note that query sequences are necessarily part of the proteome and are colored in red. (5) The statistics of each motif are k: number of occurrences in the query, l: number of occurrences in the proteome, p: number of sequences in the query, b: number of sequences in the proteome. These are written in a tabulated file used to evaluate the overrepresentation of each motif (6). The *P-value* reflecting the overrepresentation in the query set relative to the background is calculated by the cumulative hypergeometric distribution (see material and methods).

Figure 1 outlines the main procedures involved in running MotifHound. Initially the method requires two input datasets: a background set of *b* protein sequences and a query set of *q* sequences that are necessarily a subset of the background. MotifHound enumerates all possible motifs present in the query, and then computes the number of occurrences of each motif in both the query and the background sets. The unusual representation of a motif in the query compared to the background is calculated by the cumulative hypergeometric distribution. Below we describe these procedures in more details.

### Motif enumeration

The first step consists of exhaustively enumerating all possible linear motifs present in the query sequences. This is achieved by scanning the query sequences and indexing all *n*-mers, where *n* is the motif length (here composed of 3 to 12 residues). For a given *n*, the maximal size of the index is given by:
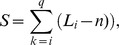
where *L_i_* is the length of the *i^th^* sequence of the query set containing *q* sequences.

Each *n*-mer from this index is then “*degenerated*”, *i.e. w* wildcards are introduced in all combinations and numbers, with the only exceptions being that (i), the first and last positions are kept constant, and (ii), we impose a minimum of three non-wildcard positions. Thus, *WC* = *C*(*n*-2,*w*) combinations of wildcard positions are explored per *n*-mer, and the occurrence of each degenerate *n*-mer is also indexed. Ultimately the maximum size of the enumerated space is *min*(*S* x *WC*, 20*^n^*). In order to speed-up the following scoring step, we retain only degenerate *n*-mers that occur at least in 3 sequences of the input set. Also, for each degenerate form of the *n*-mers, if the replacement of an amino acid by a wildcard does not add more than one occurrence, the corresponding degenerate *n*-mer is discarded. All the degenerate n-mers so obtained from the query sequences are then searched for in the background sequences and their number of occurrences is also recorded. At the end of the enumeration process, the algorithm returns an exhaustive list of all degenerate *n*-mers observed three or more times in the query sequences, along with their number of occurrences in the background sequences. In the following sections we call each degenerated *n*-mer a “motif”.

### *P*-value of motif overrepresentation in the query set

The second step consists of evaluating the biological significance of each motif by proxy of overrepresentation. Our scoring function uses the cumulative hypergeometric distribution to calculate the probability *p* to see by chance a motif present at least *k* times in *q* sequences sampled from a background set of *b* sequences and containing *l* occurrences of the motif:
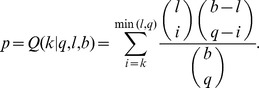
In contrast to most other algorithms, the overrepresentation of motifs calculated by MotifHound does not depend on the nature of a motif in terms of its composition or length. The influence of such properties is indeed implicitly taken into account by the number of occurrences in the background, as illustrated by the “SSSS” motif given as an example in the introduction section.

Because the exhaustive enumeration carried out favors degenerate motifs, we introduce a *P*-value adjustment. To illustrate this problem, let's compare the space of motifs of length 3 with 3 defined positions (20^3^ = 8000 possible motifs) to the space of motifs of length 10 with 3 defined positions. The latter corresponds to 20^3^×8 = 64 000 possible motifs, where 8 = C(8,7) represents all combinations for placing the third defined character in the motif (*i.e*., DDxxxxxxxD, DxDxxxxxxD, …, DxxxxxxxDD). The larger combinatorial space associated with longer motifs increases the probability of calculating a low *P-value* by chance (*i.e.* multiple-testing problem). Thus, we adjust the *P-value* according to the combinatorial space of wildcard positions the motif is associated to. The *P-value* adjustment is given by:
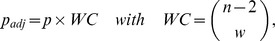
and where *p* is the *P-value* of the motif, *n* is its length, *w* is its number of wildcard positions and *WC* is the number of combinations of wildcard positions in the motif. At the end of the scoring process, the algorithm returns a file containing a list of motifs ranked by their adjusted *P-values*.

### Output for the end user

For convenience and to help in the interpretation of the results, we implemented modules that can filter the sequences to mask homologous and/or structured regions before the enumeration. It can indeed be useful to mask globular regions, because intrinsically disordered regions are known to frequently coordinate regulatory events [35], are enriched in short linear motifs [22], [36], [37], are involved in rewiring protein interactions network [38] and are often targeted for post-translational modifications [39]–[43]. Hence, we provide the option to mask ordered regions using Disopred [44], although any algorithm such as IUPred [45] or FoldIndex [46] may be used as well. Since we are interested in functional instances of LMs that arose through convergent evolution, it is also important to be able to mask homologous regions in protein sequences. To this end, we use both a filter based on BLAST [47] and another filter based on PFAM domains [24]. Note that we implemented the PFAM-based filter after the scoring of motifs as it would otherwise mask too many regions. Ultimately, users have access to the following formatted outputs:

- The HTML output (optional) consists of a table that lists the top 100 non-overlapping motifs of each length, in the query set. For each motif, the following information is given: the numbers of occurrences in the query set and in the proteome, the *P-value*, the gene name and the gene description (or the sequence ID if the gene name is unknown), the domain IDs, the description of the domain and its positions in all the sequences where it appears. The HTML output requires pre-computed information including disorder, PFAM annotations, and a flat file with the gene descriptions. We provide pre-computed information for both *S. cerevisiae* and *H. sapiens*.

- The Text output (default output and always generated) includes a list of all motifs found in the query set. For each motif, the values associated correspond, respectively, to the number of occurrences in the query and in the background, to the total number of sequences in the query and in the background and finally to the *P-value*.

### Implementation

Our algorithm has been developed with the following objectives: (i), to be able to deal with large datasets, (ii), to be user-friendly, (iii), to be organized into independent modules (*e.g.* sequence selection, homology and disorder sequence filtering, enumeration of all motifs from a set of sequences, scanning sequences with a list of enumerated motifs, computing the overrepresentation from a list of enumerated motifs and number of occurrences) and (iv), to be CPU efficient. Given these objectives, part of MotifHound is implemented in Perl for I/O error checking, enabling options and defining input parameters, manipulating sequences, taking into account additional data such as disorder predictions or BLAST results, or writing HTML output. On the other hand, the C and C++ language was used for the CPU-intensive steps, *i.e.* enumeration of motifs, scanning the background, and computing the *P-values* using the hypergeometric cumulative distribution. To optimize memory usage, a dynamic associative array was used, where each key is a motif mapped to the relevant stored numerical values (occurrences and number of sequences in both query and background, Hypergeometric *P-*value and the adjusted *P*-value, number of wildcard combination). Our method has been tested on UNIX operating systems, specifically on the most recent Ubuntu distributions (superior to 10.04 and Mint 14). A parallelized multi-thread version (x10 faster on a twelve cores machine) has been implemented but was not used for the benchmark in order to make the comparison of running times fair.

### Benchmark design

In order to compare different existing algorithms, we developed a benchmark using datasets designed for this task. To have a trustworthy comparison, we created datasets matching the input requirements of each algorithm, and measured the ability of each method to recover the motifs spiked-in. Our benchmark uses the proteome of *S. cerevisiae* as background. To reduce calculation time, we only kept sequences of 100 to 500 residues in length. Our background model is thus composed of 3,000 sequences, distributed over 30 sets of 100 randomly selected non-redundant and non-homologous sequences. For all sequences, the average length was 296±112 amino acids and the total number of amino acids was 888,505. The benchmark datasets were designed by varying three major characteristics of LMs: length *n* (varying from 3 to 10), number of wildcard positions *w* (varying from 0 to n-3) and number of occurrences *N* (taking the following values: 3, 4, 5, 6, 7, 8, 10, 12, 15, 20 and 30).

**Figure 2:**
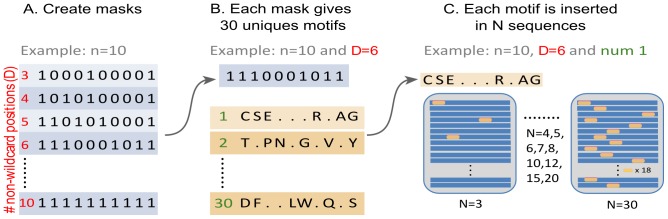
Design of the benchmark datasets. The benchmark is composed of 11880 sets of 100 sequences, each set containing a specific motif spiked-in. The motifs spiked-in vary in terms of the three following parameters: their length, n, varying from 3 to 10 residues (8 values); their number of non-wildcard positions, D, varying from 3 to n (n-2 values per length, 36 values in total); and their number of occurrences in the set, N, equal to 3, 4, 5, 6, 7, 8, 10, 12, 15, 20 or 30 (11 values). For each combination of n, D and N, we created 30 replicates varying in the motif being spiked-in. Altogether, 1080 motifs were created for the benchmark (30 replicates ×36 masks), resulting in 11880 sets of 100 sequences (1080 motifs ×11 number of occurrences). **A**. We first create masks for each motif length in order to assign the wildcards and non-wildcard positions. ‘Ones’ indicate non-wildcard positions and ‘zeros’ indicate wildcard positions. The first and last positions are always non-wildcard, thus, *n*-2 masks are created for each length *n*, yielding 8+7+6+5+4+3+2+1 = 36 masks for lengths 10 to 3. **B**. In the second step, each mask is used to derive 30 unique motifs, by shuffling all positions (except the first and last) and replacing all non-wildcard positions by amino acids with frequencies drawn from the yeast proteome. In this example, 30 unique motifs are generated from a mask containing D = 6 non-wildcard positions. **C**. Finally, each motif so obtained is spiked-in once in *N* sequences from a set, each composed of one hundred yeast protein sequences randomly sampled. The orange rectangles symbolize the motifs spiked in, and the blue lines represent sequences. In this example, the motif has been inserted either 3, 4, 5, 6, 7, 8, 10, 12, 15, 20 or 30 times in the same dataset of 100 sequences.

**Figure 3:**
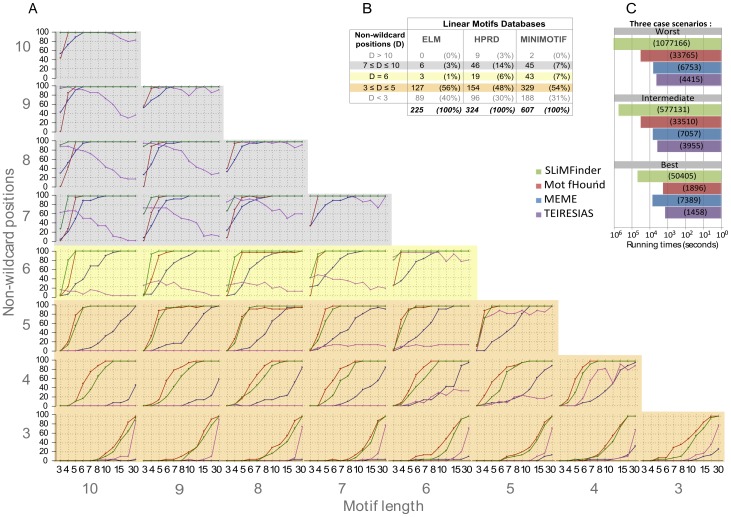
Comparative analysis of different algorithms in the discovery of degenerate linear motifs. A. Each graph shows the motif detection accuracy (y-axis) as a function of the number of sequences *N* where the motif was spiked-in (x-axis, number 12 and 20 are not shown). Each graph shows the results for a motif of length *n* (columns) and with 3 to 10 non-wildcard positions (*i.e.* 0 to 7 wildcards) (rows). Colors correspond to the methods tested (red: MotifHound, blue: MEME, green: SLiMFinder and purple: TEIRESIAS). The accuracy is assessed by the capacity of each method to recover the motifs spiked-in the datasets. For each combination of parameters (length, number of wildcards, number of occurrences), there are 30 unique motifs inserted, each in a unique set of 100 sequences. Thus, each motif correctly identified increases the accuracy by 3.33%, and an accuracy of 100% means that the motifs in all 30 replicates were correctly identified as being the most significant. **B**. Statistics on length and number of wildcard positions for biological motifs extracted from the Human Protein Resource Database [33] (HPRD), the Eukaryotic Linear Motif database [32] (ELM) and the MiniMotif resource [34]. We present the statistics for 5 groups of motifs defined according to the number D of non-wildcard positions that they contain (D<3; 3≤D≤5; D = 6; 7≤D≤10; D>10). **C**. Barplot of running times in seconds for three case-scenarios representing different levels of motif-search complexity. The best scenario is a short well-defined motif (length 4 and 1 wildcard), the worst scenario is a long and degenerate motif (length 10 and 6 wildcards), and an intermediate scenario corresponds to a long and little degenerate motif (length 10 and 3 wildcards). Each color corresponds to a method, as in (A).

In order to create the motifs spiked-in, we proceeded in two steps as described in Figure 2. In the first step, we created 36 masks representing the number of wildcards and defined positions: 8 masks for the length 10, 7 masks for the length 9, and so on, until 1 mask for the length 3. Each mask was then used to derive 30 unique motifs generated, first by shuffling the positions of wildcards, and second by replacing the defined positions with amino acids. Finally, each unique motif was inserted in a set of 100 sequences, with one motif at most per sequence. To vary the number of occurrences, each motif was inserted different number of times (3, 4, 5, 6, 7, 8, 10, 12, 15, 20 and 30) in replicates of the same sequence set. Altogether, the complete benchmark dataset was composed of 11,880 sets of 100 sequences (30 unique motifs ×11 occurrence numbers ×36 masks). Thus, each set contained a single motif of length *n* spiked-into *N* sequences. To compute an accuracy value for each set of 100 sequences, the top (most significant) motif found by each algorithm was kept and compared to the motif inserted in the set. Thirty sets were created for each combination of parameters (*n*, *w*, *N*). The accuracy shown in Figure 3 thus corresponds to the fraction of sets where the correct motif was identified, *i.e.* “Number of correct identifications divided by 30”.

### Binding energy, solvent accessibility, evolutionary conservation and intrinsic disorder of motifs predicted to bind FUS1 SH3 domain

Binding energies were calculated based on scoring matrices from Fernandez-Ballester *et al*. [48], and available in the ADAN database [49]. Solvent accessibility was predicted by SABLE version 2 [50]. In our experiments, only residues with highest score (*i.e.* equal to 9) of solvent accessibility were considered in the analysis. To calculate evolutionary conservation, we retrieved homologues by PSI-BLAST using the default NR database from NCBI [51], imposing a minimum of 35% sequence identity. Redundancy among homologues was filtered by CD-HIT [52] using 95% maximum identity. Sequences kept were aligned using MUSCLE [53] and evolutionary rates of individual amino acids were calculated with Rate4Site [54]. Protein disorder was predicted using DISOPRED2 [55]. Only residues with the highest score of intrinsic disorder (*i.e.* equal to 9) were considered as disordered.

## Results and Discussion

Several algorithms have been developed to help in the discovery of linear motifs. We summarize some of their key features in Tables **Table 1**. DILIMOT, Fire-Pro, NestedMica, ANCHOR, SLIMPRED, qPMS7, D-STAR, and phylo-HMM were not utilized in the benchmark for different reasons described in the table. However all these algorithms are below in the identification of linear motifs experimentally determined to bind to the FUS1 SH3 domain from *S. cerevisiae*. We present the results of the four methods in Figure 3, and we provide details the parameters used to run the different programs in Tables S1 and S2 in File S1. Note that some algorithms would have required too many CPU-hours, and were thus applied to a subset of the benchmark. Results for these are shown in Figures S1 and S2 in File S1. The four methods for which results are shown in Figure 3 are MEME, MotifHound, SLiMFinder and TEIRESIAS.

### Benchmark design

It is difficult to evaluate the performance of motif discovery tools, as no large-scale annotated and curated dataset is available for this task. Moreover, while the sequence of a specific motif may be known, the functional relevance of all its instances in biological sequences remains unknown. In order to analyze how different algorithms behave with motifs exhibiting different properties, we designed a benchmark that comprehensively explores three major characteristics of LMs. The benchmark design was achieved by inserting artificial motifs in protein sequences extracted from the budding yeast (*S. cerevisiae*) proteome. Such design implies that there is no simulated data beyond the motifs randomly spiked-in. The sequences were indeed not simulated, as we used biological sequences from yeast, which come with all their natural features (*e.g.* tandem-repeats and low complexity regions).

One advantage of such a setup is that it enables us to assess the individual contribution of different properties on the probability to accurately identify a motif. The motifs spiked-in varied according to the 3 following properties: (i), length, (ii), number of defined positions and (iii), overrepresentation (*i.e.* number of sequences out of the one hundred in each set that was spiked-in with a motif). For each combination of these three parameters, 30 different artificial motifs were introduced into 30 sets, yielding a total of 11,880 sets. Accuracy was computed by the percentage of sets in which the artificially inserted motif was correctly identified (material and methods). We show the results of the benchmark in Figure 3. Each graph shows the accuracy of motif identification (y-axis) as a function of the number of sequences where the motif was inserted (x-axis). The graphs are displayed such that the first column shows the results for motifs of size 10, the second for motifs of size 9, *etc*. In parallel, the first row shows the results for 10 defined positions (0 wildcards), the second for 9 defined positions (1 wildcard), *etc*. We thus compare the performance of the four algorithms tested as a function of both: motif degeneracy (columns) and motif size (rows).

### Accuracy increases with information content and number of occurrences

Globally, we see a similar and expected trend for all methods: given a motif, the more occurrences are spiked-in (x-axis on each graph, Figure 3A) and the more information it contains (bottom row to top row), the more frequently it is accurately discovered. We broadly identify three regimes of accuracy that are strongly dependent on the number of non-wildcard positions.

First, for 3 non-wildcard positions, a minimal number of occurrences *N*∼20 is required to identify more than 75% of inserted motifs. Interestingly, all other parameters being equal, a small motif can be more reliably identified than a large one. For example, motifs of length 3 or 4 can be correctly identified in 80% of cases when only 15 occurrences are spiked-in. Thus, given a constant number of defined amino acids in a motif, the addition of wildcards decreases the probability to detect it accurately. The addition of wildcards indeed increases the space of “negative” motifs, which likely blurs the signal of the motif spiked-in. For example, considering a motif of length 3 and a motif of length 10, both containing 3 non-wildcard positions, the motif of length 3 leads to a maximum of 20^3^ (8000) possibilities of distinct motifs whereas that of length 10 is associated with a space 8 times larger (64000).

Second, considering 4 non-wildcard positions, a minimal number of occurrences *N* = 7 is needed to accurately identify the motif in more than 75% of the sets. Thus, adding a single defined amino acid to the motifs drastically reduces the minimum number of occurrences required to accurately identify them.

Finally, in the third regime we found motifs that comprise at least 5 non-wildcard positions. For those we observed a point (*N* = 5), beyond which, most accuracy curves quickly reach 100%. Overall, the benchmark delimits the boundaries of the twilight zone by providing baselines regarding the minimal features that linear motifs should exhibit in order to be detectable by overrepresentation alone.

In the description above we discussed the results obtained using MotifHound. The results of SLiMFinder are overall similar, and mostly depend on the information content of a motif: when the information content is low (*i.e.* 3 to 5 defined positions), MotifHound finds the motifs spiked-in at a slightly though consistently higher accuracy than SLiMFinder, however, as information content increases, SLiMFinder exhibits higher accuracies when low numbers of repeats of the motifs are present. The performance of TEIRESIAS and MEME, on the other hand, are more dependent on the information content of motifs. For example, for motifs of length 10 and inserted 6 times in 100 sequences, more than 90% of them are found by all methods when there is no more than one wildcard position, but only ∼50% (MotifHound), ∼20% (SLiMFinder) and ∼5% (TEIRESIAS and MEME) of them are found when their number of defined positions drops to 4.

The accuracies obtained with TEREISIAS can appear surprising in that accuracy can drop as more occurrences of the motifs are introduced. The reason for this behaviour is the scoring function of TERESIAS, which is sometimes not accurate. Specifically, as more copies of a motif are spiked-in, it increases the likelihood that a variation of the motif introduced receives a better score.

Overall, for motifs containing only 3 to 5 defined amino acids, MotifHound and SLiMFinder tend to perform better than MEME and TEIRESIAS. Yet, for motifs with 6 or more defined positions, SLiMFinder is more sensitive for their discovery at low numbers of occurrences (*N* = 3, 4 or 5 in 100 sequences).

### Degenerate linear motifs are frequent in proteins and are best detected with MotifHound

We show the distribution of known functional motifs retrieved from three databases (HPRD, ELM and MiniMotif) according to their number of non-wildcard positions (Figure 3B). A non-wildcard position was counted when it was defined by two amino acids at most (*e.g.* S/T or D/E). Conversely, a wildcard position was counted when at least 3 different amino acids were tolerated at a given position. This shows that about half of known functional motifs described in these 3 databases contain only 3 to 5 non-wildcard positions. Consistently, we know that only a few (∼1/3) hotspot residues are conserved in linear motifs [2], [10]. For this type of degenerate motif, representing 50% of known functional motifs (yellow background, Figure 3A and Figure 3B), MotifHound performs best compared to other existing methods. Among motifs with higher information content (from 6 to 10 non-wildcard positions), representing ∼15% of known functional motifs, SLiMFinder outperforms MotifHound. The advantage of MotifHound that we saw earlier, *i.e.*, that it does not take into account the structure of the motif itself but only its distribution of occurrences, is thus also its weakness in this particular scenario.

Overall, for most biologically relevant motifs with low information content (3 to 5 non-wildcard positions), the scoring function of MotifHound (cumulative hypergeometric distribution) is able to discriminate the motif spiked-in better than other existing approaches. This makes it a promising approach to discover new linear motifs mediating protein-protein interactions involved in cell signalling and regulation, as these usually exhibit low information content (Figure 3B).

### Running time

The barplot in Figure 3C, shows three scenarios in terms of running time: worst-case scenario (motifs of length 10 with 4 defined positions), best-case scenario (motifs of length 4 with 3 defined positions) and an intermediate scenario (motifs of length 10 with 7 defined positions). In each scenario and for each method, 330 benchmark datasets were processed. Regarding MEME, running times are stable and do not depend on the case scenario. The MEME's average running time for one dataset is about 16 seconds on the workstation we used.

In the best-case scenario, MotifHound competes with TEIRESIAS, but it significantly slows down in the intermediate and worst case scenario. On average, for the length 4, MotifHound can process a dataset in 5 seconds. For length 10 however, it takes about 100 seconds for both the intermediate and worst-case scenarios (note that in MotifHound, running times are impacted by the motif length and not by the ratio of defined to wildcard positions in the motifs). TEIRESIAS is the fastest method in all scenarios even though running times increase for longer motifs. On average, for the length 4, it takes 4 seconds to process each dataset and it increases to only 13 seconds for the length 10. Considering SLiMFinder running times, 440 seconds are required in the best-case scenario, 1758 seconds in the normal case scenario, and 5920 seconds in the worst-case scenario.

In Table 2, we compared running times for all methods and for all the benchmark datasets. Running times of all methods are affected by the motif's length, but also because increasing the motif length requires more benchmark sets in order to cover all combinations of *n*, *w*, *N*. Overall, MotifHound is ∼17 times faster than SLiMFinder, ∼2 times slower than MEME, and ∼9 times slower than TEIRESIAS. Interestingly, it is very fast for short motif lengths, remaining faster than MEME up to a length of 6 amino acids. The complexity of our algorithm indeed scales quadratically with the motif length because we explore the complete combinatorial space of degenerate motifs.

**Table 2:** Run times for the main methods used in the benchmark.

Motifs Length	*Number of Sets*	Total running time in minutes			
		TEIRESIAS	MEME	SLiMFinder	MotifHound
**3**	*330*	26	113	140	21
**4**	*660*	57	251	774	83
**5**	*990*	91	376	2808	207
**6**	*1320*	129	476	7869	417
**7**	*1650*	170	608	18632	796
**8**	*1980*	215	740	37153	1645
**9**	*2310*	255	863	58457	3096
**10**	*2640*	493	1003	80689	5866
**Total**	***11880***	**1436**	**4430**	**206522**	**12133**

### Application to the identification of motifs binding to the FUS1 SH3 domain

The yeast protein FUS1, which is involved in the mating process, contains a SH3 domain that has been shown to bind to 25 peptides (binding sites) within 22 different proteins [56], [57]. Among the 25 known binding sites (Table S3 in File S1), some include the consensus motif R[ST][ST]SL and others do not. We compared different algorithms in the identification of the 25 experimentally characterized SH3 binding sites. To this end, we used the same algorithms as in the benchmark, three webservers (DILIMOT, SLIMPRED, and qPMS7) and three additional algorithms, ANCHOR, SLIMPRED and phylo-HMM, which aim at detecting motif-containing regions rather than specific motifs. The parameters used for each algorithm are detailed in the supplementary material (Table S4 in File S1). For each algorithm we selected the top scoring motifs or regions such that they covered 2.15% of the total length of the 22 protein targets, a coverage that is equivalent to that of the 25 binding sites in the 22 protein sequences. The coverage could however be smaller than 2.15% if the algorithm did not return enough motifs, or could be larger if a large number of motifs were returned with identical scores, as with D-STAR.

**Figure 4:**
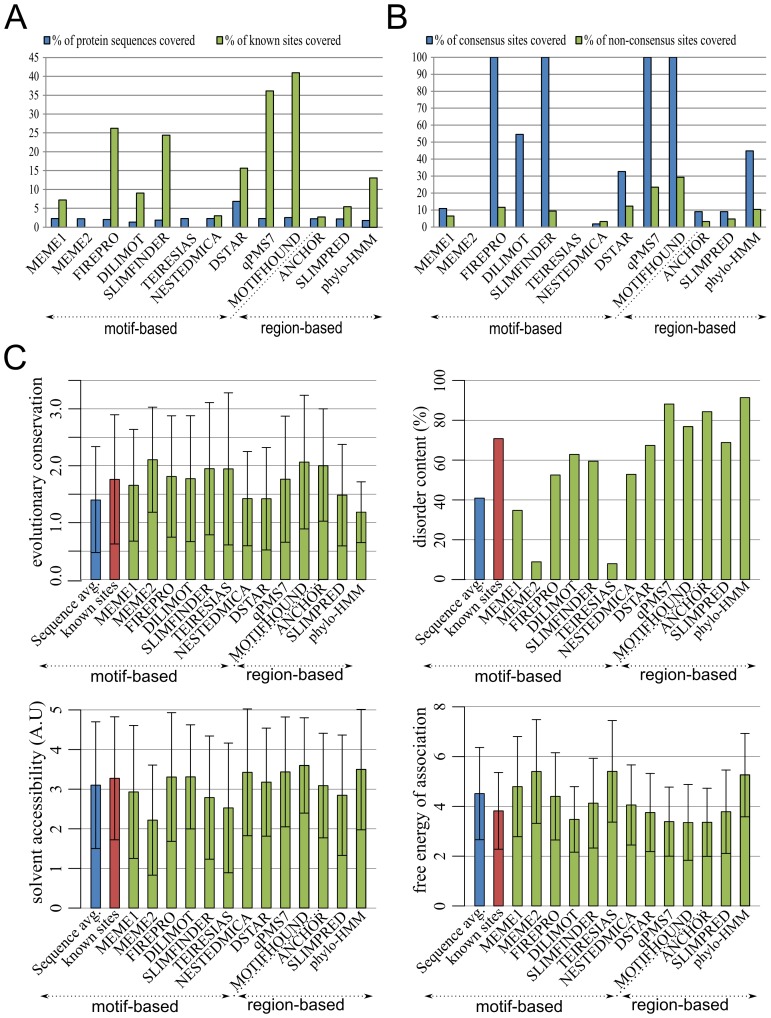
Detection of linear motifs in 22 protein targets known to bind the FUS1 SH3 domain. **A**. FUS1 is a yeast protein involved in mating. The SH3 domain of FUS1 is known to bind 22 proteins, through 25 binding sites that cover 2.15% of their total sequence length. We blindly submitted the 22 protein sequences to several algorithms for them to detect the binding sites. Two types of algorithms were considered, motif-based algorithms, which detect specific overrepresented motifs, and regions-based algorithms, which detect regions predicted to encode any linear motif. We then considered the top motifs (or regions) returned for each algorithm, such that they covered at most 2.15% of sequence's length. For each algorithm tested, we plot the coverage of the motifs identified, as well as the corresponding coverage of known SH3 binding sites identified. **B**. Among motifs experimentally characterized to mediate the recognition between FUS1 and its partners, some motifs correspond to the known consensus R[ST][ST]SL and others do not. Here we plot the fraction of coverage for both types, showing that MotifHound is particularly able to identify non-consensus sequences. Together, the results in panels A and B show that the measure of “*motif enrichment*” introduced with MotifHound enables the accurate detection of functional linear motifs, and is in fact the best in this case. **C**. We know that linear motifs tend to exhibit specific biological signatures. They indeed tend to be conserved, they tend to appear in solvent-accessible as well as in disordered regions, and in the case of SH3 recognition motifs they should exhibit favourable free energies of association with the SH3 domain. We compared these properties for motifs identified using the different methods, showing that those returned by MotifHound consistently reflect these properties.

For each algorithm, we show in Figure 4A the percentage of the total length of the 22 sequences covered by the motifs identified (blue bars), as well as the percentage of the total length of known SH3 binding sites covered (green bars). We found that the motifs predicted by FIRE-Pro, SLiMFinder, qPMS7, and MotifHound exhibit the largest overlap with experimentally characterized binding sites, with an advantage to MotifHound. In Figure 4B, we show the coverage of each algorithm for binding sites that correspond to the consensus R[ST][ST]SL motif (blue), or that do not (green bars). We found that, FIRE-Pro, SLiMFinder, qPMS7, and MotifHound predicted all binding sites corresponding to the consensus R[ST][ST]SL, yet only qPMS7 and MotifHound were best at identifying other binding sites with ∼23% and ∼30% coverage respectively.

Next, we examined the motifs identified by the different methods in terms of properties generally expected of SH3 binding motifs (high evolutionary conservation, high solvent accessibility, high intrinsic disorder, and low free energy of association with their cognate partner). In Figure 4C, we show these properties for known (red) and predicted (green) SH3 binding sites. Remarkably, the figure reveals that motifs identified by MotifHound exhibit the expected signature for these properties despite the fact that no such information was used in the first place. These results therefore illustrate that scoring motif enrichment by the strategy employed in MotifHound is effective and complementary to other methods.

## Conclusion

To conclude, MotifHound exhibits a good combination of speed and accuracy. The accuracy is indeed comparable to that of SLiMFinder and even higher for degenerate motifs, while the speed is comparable to that of MEME and sometimes higher for short motifs. Moreover, the benchmark we carried out provides lower estimates of motifs statistics required for their discovery, *i.e.* we observed that identification of motifs is accurate when they are defined by more than 4 amino acids and occur in over 6 sequences out of 100. The validity of our approach has been reinforced by the correct identification of linear motifs characterized to bind to a SH3 domain. The robust and simple framework upon which MotifHound is based, together with the fact that it is developed as an open-source project, will make it a solid platform for future research involving discovery of linear motifs mediating new functions, to ultimately better understand protein-peptide interactions [58] and open new possibilities for drug design [59].

## Supporting Information

File S1 **Supplementary Tables S1–4 and supplementary Figures S1–2.**
(DOCX)
