# Supplementary material for: Fast and Accurate Discovery of Degenerate Linear Motifs in Protein Sequences
Source: PLoS One. 2014 Sep 10;9(9):e106081. doi: 10.1371/journal.pone.0106081 (PMC4160167; doi:10.1371/journal.pone.0106081)
Supplement: File S1 — Supplementary Tables S1–4 and supplementary Figures S1–2. (DOCX) [file pone.0106081.s001.docx]

***Supplementary material for:***

MotifHound: fast and accurate discovery of protein linear motifs

Abdellali Kelil^+^, Benjamin Dubreuil^+^, Emmanuel D. Levy^*^ and Stephen W. Michnick^*^

**Table S1. Parameters used for the benchmark with the different programs tested**

| Methods | Motif Discovery parameters |
| --- | --- |
| Fire-pro | -expfile=**SeqExp** -species=**yeast** -exptype=**discrete** -kmers=**3-*S*** -gaps=**0-(*S*-*C*)** |
| MotifHound | -Setfile=**SeqFasta** -Proteome=**SeqBack** -Size=***S*-*S*** |
| MEME | **SeqFasta** -mod=**zoops** -protein -bfile=**MarkBack.ord2**  -nmotifs=**1** -minsites=**3** -maxsites=**30** -maxiter=**100 -w=S** -nomatrim |
| SLiMFinder | -seqin=**SeqFasta** -aafreq=**MarkBack.ord0** -minwild=**0** -maxwild=**min(*S*-*C*,4)** -slimlen=***C*** -eFilter=**F**  -minocc=**3** -walltime=**3.0** -termini=**F** -extras=**F** -savespace=**2** -preamb=**F** |
| TEIRESIAS | -l=***C*** -w=***S*** -c=**0** -n=**0** -q=**100** -k=**3** -v -u -p -s |
| Nested MICA | -alphabet **protein** -maxLength ***S*** -minLength ***S*** -seqs **SeqFasta**  -backgroundModel **MarkBack.ord2** -numMotifs **1** |

***S*** = Length of the motif,

***C*** = Number of non-wildcard positions,

**SeqExp** = Expression profile for 100 randomly selected protein sequences,

**SeqFasta** = Sequence file containing 100 randomly selected protein sequences in FASTA format,

**MarkBack.ord*X*** = Markov Background of order ***X***

Below we outline general as well as method-specific parameters used in this study:

**Table S2. Parameters that are method specific**

| Methods | Parameter | Description |
| --- | --- | --- |
| FIRE-pro | -expfile | Input expression profile ( all proteins are listed and associated to a value) |
|  | -species | Name of the organism where the sequences should be extracted from |
|  | -exptype | Type of value in the expression profile ( in that case, a binary value, 0 for no expression and 1 for expression) |
|  | -kmers | Lower and Upper bound for the length of kmers in the k-mer exploration step |
|  | -gaps | Lower and Upper bound for the number of gaps (wildcard inserted in the middle of the k-mer seed) |
| **MotifHound** | -Setfile | Query set containing the protein sequences of interest |
|  | -Proteome | Background set |
|  | -Size | Lower and Upper bound for the length of motif in the search step |
| **MEME** | Standard input | Set of sequences in which motifs are searched for |
|  | -mod | Model of motifs distribution (zoops = zero or one per protein sequence) |
|  | -protein | Sequences use protein alphabet |
|  | -bfile | Background file in the form of nth-order Markov chain |
|  | -nmotifs | Maximum number of motifs to find |
|  | -w | Width of motifs |
|  | -minsites | Minimum number of sites for each motif |
|  | -maxsites | Maximum number of sites for each motif |
|  | -maxiter | Maximum Expectation-Maximization iterations to run |
|  | -nomatrim | Do not adjust motif width using multiple alignments |
| **SLiMFinder** | -seqin | Sequence file to search |
|  | -aafreq | Use a model to replace individual sequence Amino Acid Frequencies (here a Markov chain of order 0) |
|  | -minwild | Minimum number of consecutive wildcard positions to allow |
|  | -maxwild | Maximum number of consecutive wildcard positions to allow |
|  | -slimlen | Maximum length of motifs to return (Number of non-wildcard positions) |
|  | -minocc | Minimum number of unrelated occurrences for returned motifs. (Proportion of UP if < 1) |
|  | -efilter* | Whether to use evolutionary filtering |
|  | -termini* | Whether to add termini characters (^ & $) to search sequences |
|  | -walltime* | Time in hours before program will abort search and exit |
|  | -preamb* | Whether to search for ambiguous motifs during motif discovery |
|  | -extras* | Whether to generate additional output files (alignments etc.) |
|  | -savespace* | Delete "unnecessary" files following run (best used with tar.gz):  0 = Delete no files,  1 = Delete all bar *.upc and *.pickle files,  2 = Delete all dataset-specific files (*.upc and *.pickle and not *.tar.gz) |
| **TEIRESIAS** | -l | Number of non-dot characters in the pattern |
|  | -w | The maximum extent of an elementary pattern (Length of a motif) |
|  | -c | Number of overlapping in the convolved pattern |
|  | -n | Number of maximum brackets allowed in a pattern |
|  | -k | Minimum allowed support (occurrences) for a pattern |
|  | -q | Maximum allowed support (occurrences) for a pattern |
|  | -v | If given, then -k is the minimum number of sequences in which a pattern should appear |
|  | -u | Consider only the uppercase characters during pattern discovery |
|  | -p | Output the list of positions (offset list) for each discovered pattern |
|  | -s | Do scanning and output the sample list only (elementary patterns |
| **NestedMICA** | -seqs | Sequence file to search |
|  | -backgroundModel | Background file in the form of nth-order Markov chain (here, order 2 with 5 classes) |
|  | -alphabet | Sequences use protein alphabet |
|  | -minLength | Minimum length of motif |
|  | -maxLength | Maximum length of motif |
|  | -numMotifs | Maximum number of motifs to find |

****Those following parameters have been disabled in order to reduce the running times without affecting the accuracy.***

Table S3. List of regions known to bind FUS1 SH3 domain that were used in the case study. Note that amino acids that are part of overlapping regions were only counted once in our analysis.

| ORF | Gene ID | Binding Region | | Reference |
| --- | --- | --- | --- | --- |
|  |  | *START* | *END* |  |
| YIL159W | *BNR1* | 639 | 653 | [[1](#_ENREF_1)] |
| YNL042W | *BOP3* | 291 | 305 | [[1](#_ENREF_1)] |
| YGL116W | *CDC20* | 43 | 57 | [[1](#_ENREF_1)] |
| YPR023C | *EAF3* | 194 | 208 | [[1](#_ENREF_1)] |
| YDR206W | *EBS1* | 627 | 641 | [[1](#_ENREF_1)] |
| YLR056W | *ERG3* | 80 | 94 | [[1](#_ENREF_1)] |
| YGR098C | *ESP1* | 1127 | 1141 | [[1](#_ENREF_1)] |
| YKR049C | *FMP46* | 6 | 20 | [[1](#_ENREF_1)] |
| YCL039W | *GID7* | 107 | 121 | [[1](#_ENREF_1)] |
| YAL031C | *GIP4* | 527 | 541 | [[1](#_ENREF_1)] |
| YPR008W | *HAA1* | 497 | 511 | [[1](#_ENREF_1)] |
| YPL224C | *MMT2* | 26 | 40 | [[1](#_ENREF_1)] |
| YPL070W | *MUK1* | 176 | 190 | [[1](#_ENREF_1)] |
| YCL052C | *PBN1* | 40 | 54 | [[1](#_ENREF_1)] |
| YNL267W | *PIK1* | 370 | 384 | [[1](#_ENREF_1)] |
| YPL156C | *PRM4* | 133 | 147 | [[1](#_ENREF_1)] |
| YMR308C | *PSE1* | 276 | 290 | [[1](#_ENREF_1)] |
| YJL047C | *RTT101* | 745 | 759 | [[1](#_ENREF_1)] |
| YMR140W | *SIP5* | 41 | 55 | [[1](#_ENREF_1)] |
| YBR172C | *SMY2* | 75 | 89 | [[1](#_ENREF_1)] |
| YER090W | *TRP2* | 254 | 268 | [[1](#_ENREF_1)] |
| YIL159W | *BNR1* | 639 | 650 | [[2](#_ENREF_2)] |
| YIL159W | *BNR1* | 640 | 654 | [[2](#_ENREF_2)] |
| YDR103W | *STE5* | 111 | 122 | [[2](#_ENREF_2)] |
| YDR103W | *STE5* | 112 | 126 | [[2](#_ENREF_2)] |

Table S4. Usage of the algorithms tested on the 22 proteins binding partners of FUS1 SH3 domain

| Methods | Usage | Motif Discovery parameters |
| --- | --- | --- |
| MotifHound | Local | -Proteome=***S. cerevisiae* proteome** -Size=***3 to 10*** |
| SLiMFinder | Webserver | <http://bioware.ucd.ie/~compass/biowareweb/Server_pages/slimfinder.php>  Mask out ordered residues=***OFF*** Mask out unconserved residues***=OFF***  Mask out the N-terminal=***M*** Minimum disordered length for inclusion=***0***  Mask out low complexity regions=***[5,8]***  Mask out position specific residues=***2:A***  Min. #. consecutive wildcards=***0*** Max. #. consecutive wildcards=***2***  Max. # wildcards=***5*** Min. # unrelated seqs with motif=***3***  Blast expectation (E) value=***0.0001*** Find ambiguity=***OFF***  Allow variable length wildcards=***OFF***  Significance cut off for returning results=***0.99***  Probabilities based on residue frequencies after masking=***OFF***  Only return max. this no. of motifs=***100*** Min. information content of motifs=***2.1*** |
| TEIRESIAS | Local | -l=***3*** -w=***10*** -c=***0*** -n=***0*** -q=***100*** -k=***3*** -v -u -p –s |
| NestedMICA | Local | -alphabet ***protein*** -minLength ***3*** -maxLength ***10*** -numMotifs ***100***  -backgroundModel ***MarkBack.ord2*** |
| MEME1  MEME2 | Webserver | <http://meme.nbcr.net/meme/cgi-bin/meme.cgi>  minimum width=***3*** maximum width=***10***  maximum number of motifs to find=***100***  any number of repetitions***=checked***  proteins tested negatively for binding to FUS1 SH3 domain are use as negative controls with MEME2 |
| FIRE-PRO | Webserver | [https://iget.c2b2.columbia.edu](https://iget.c2b2.columbia.edu/)  Species=***Saccharomyces cerevisiae*** Distribution type=***continuous*** |
| DILIMOT | Webserver | [http://dilimot.russelllab.org](http://dilimot.russelllab.org/)  Remove=***Smart domains, Pfam domains, Homology***  Your species=***Saccharomyces cerevisiae***  Fixed positions=***3*** Maximum motif length=***10***  Minimal number of motifs in your=***100*** |
| ANCHOR | Webserver | [http://anchor.enzim.hu](http://anchor.enzim.hu/)  ***(no parameters)*** |
| SLIMPRED | Webserver | [http://bioware.ucd.ie/~compass/biowareweb](http://bioware.ucd.ie/~compass/biowareweb/)  ***(no parameters)*** |
| qPMS7 | Webserver | [http://pms.engr.uconn.edu](http://pms.engr.uconn.edu/)  The percentage of protein sequences containing motifs***=75%***  Single-box***=Any*** Full Search***=checked*** |
| D-STAR | Local | -l ***3 to 10*** -d ***2*** -L ***6*** -R ***6*** -I ***8*** -N ***100*** |
| phylo-HMM | Webserver  Predictions | <http://www.moseslab.csb.utoronto.ca/phylo_HMM/>  **(no parameters)** |

**LENGTH 4**

**
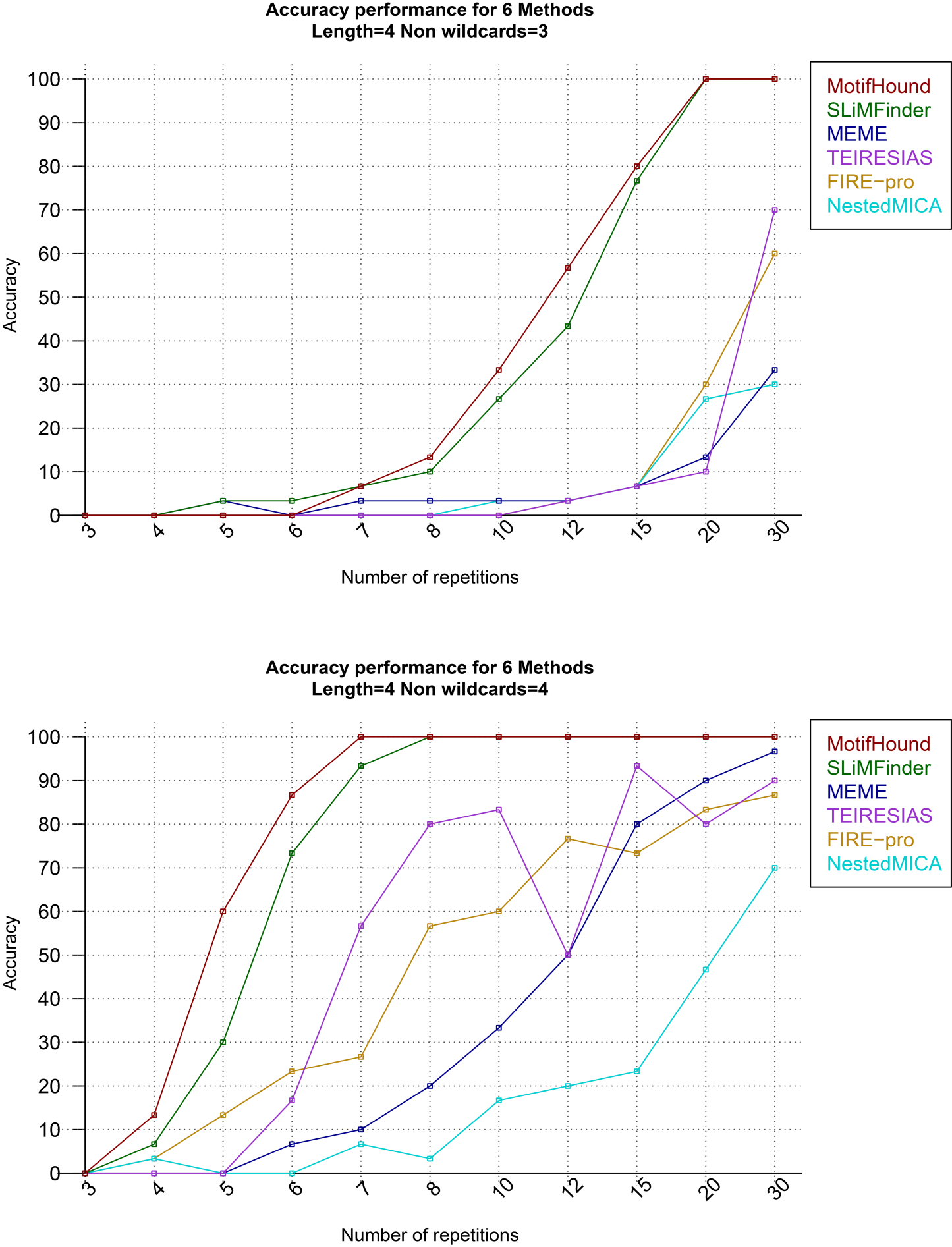
**

**LENGTH 10**

Figure S1. Results of the benchmark for motifs of length 4 or 10, with 3 and 4 defined positions respectively.


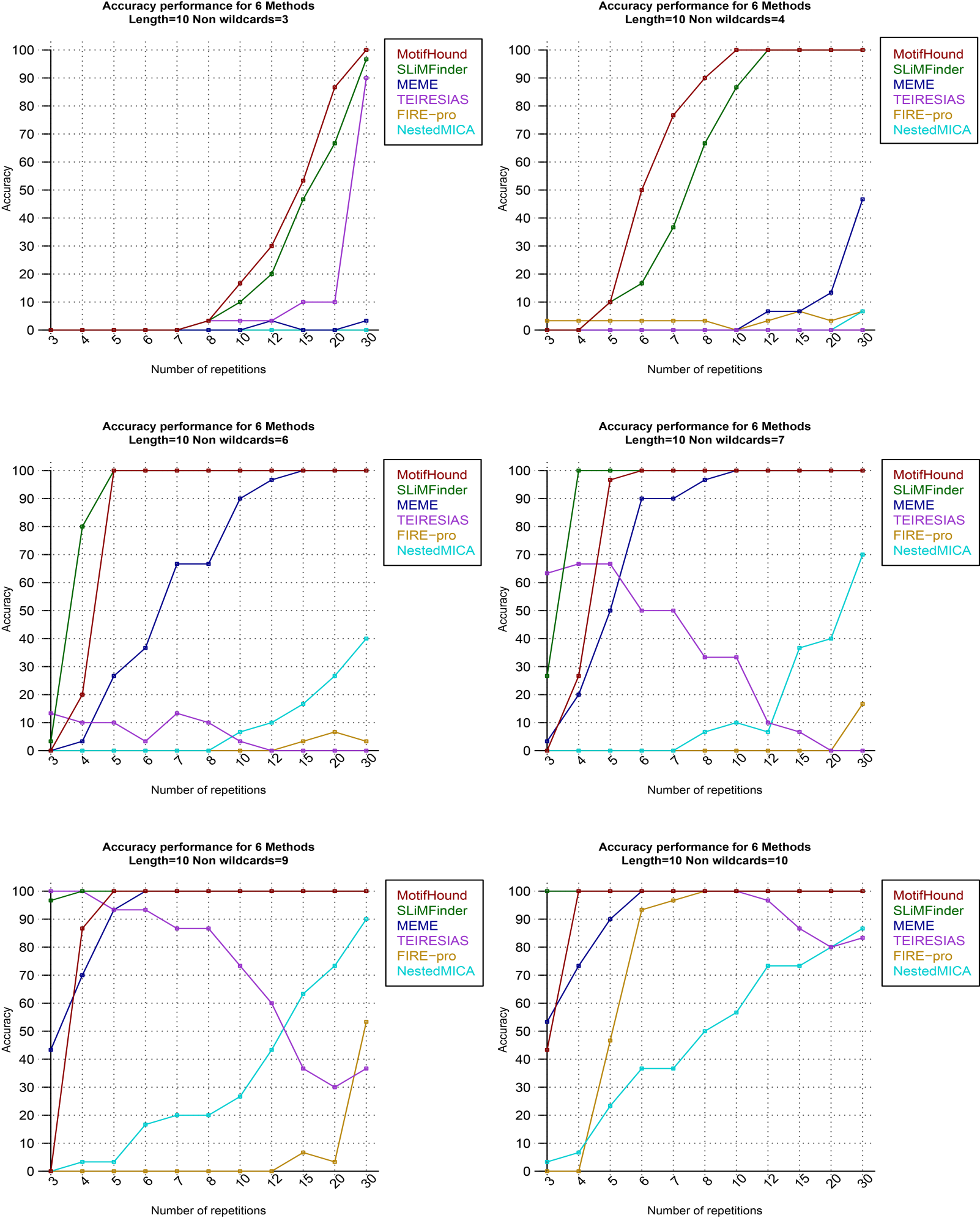


Figure S2. Results of the benchmark for motifs of length 10, with 3 or 4 (top), 6 or 7 (middle) and 9 or 10 defined positions.

1. Tonikian R, Xin X, Toret CP, Gfeller D, Landgraf C, et al. (2009) Bayesian modeling of the yeast SH3 domain interactome predicts spatiotemporal dynamics of endocytosis proteins. PLoS Biol 7: e1000218.

2. Kim J, Lee CD, Rath A, Davidson AR (2008) Recognition of non-canonical peptides by the yeast Fus1p SH3 domain: elucidation of a common mechanism for diverse SH3 domain specificities. J Mol Biol 377: 889-901.
